# Long‐term investigation of microbial community composition and transcription patterns in a biogas plant undergoing ammonia crisis

**DOI:** 10.1111/1751-7915.13313

**Published:** 2018-10-31

**Authors:** Martin Alexander Fischer, Simon Güllert, Sarah Refai, Sven Künzel, Uwe Deppenmeier, Wolfgang R. Streit, Ruth Anne Schmitz

**Affiliations:** ^1^ Institute of General Microbiology Christian‐Albrechts‐University Kiel Am Botanischen Garten 1‐9 24118 Kiel Germany; ^2^ Institute of Microbiology & Biotechnology University Hamburg Biozentrum Klein Flottbek Hamburg Germany; ^3^ Institute of Microbiology & Biotechnology University Bonn Meckenheimer Allee 168 53115 Bonn Germany; ^4^ Max‐Planck‐Institute of Evolutionary Biology August‐Thienemann‐Str. 2 24306 Plön Germany

## Abstract

Ammonia caused disturbance of biogas production is one of the most frequent incidents in regular operation of biogas reactors. This study provides a detailed insight into the microbial community of a mesophilic, full‐scale biogas reactor (477 kWh h^−1^) fed with maize silage, dried poultry manure and cow manure undergoing initial process disturbance by increased ammonia concentration. Over a time period of 587 days, the microbial community of the reactor was regularly monitored on a monthly basis by high‐throughput amplicon sequencing of the archaeal and bacterial 16S rRNA genes. During this sampling period, the total ammonia concentrations varied between 2.7 and 5.8 g l^−1^ [NH_4_^+^–N]. To gain further inside into the active metabolic pathways, for selected time points metatranscriptomic shotgun analysis was performed allowing the quantification of marker genes for methanogenesis, hydrolysis and syntrophic interactions. The results obtained demonstrated a microbial community typical for a mesophilic biogas plant. However in response to the observed changing process conditions (e.g. increasing NH_4_^+^ levels, changing feedstock composition), the microbial community reacted highly flexible by changing and adapting the community composition. The *Methanosarcina*‐dominated archaeal community was shifted to a Methanomicrobiales‐dominated archaeal community in the presence of increased ammonia conditions. A similar trend as in the phylogenetic composition was observed in the transcription activity of genes coding for enzymes involved in acetoclastic methanogenesis and syntrophic acetate oxidations (Codh/Acs and Fthfs). In accordance, Clostridia simultaneously increased under elevated ammonia concentrations in abundance and were identified as the primary syntrophic interaction partner with the now Methanomicrobiales‐dominated archaeal community. In conclusion, overall stable process performance was maintained during increased ammonia concentration in the studied reactor based on the microbial communities’ ability to flexibly respond by reorganizing the community composition while remaining functionally stable.

## Introduction

Anaerobic biomass degradation is a widely applied technique for green energy generation form organic waste or energy crops (Weiland, 2010; Maus *et al*., 2016a). During the last decades, the generation of biogas by anaerobic degradation was in the focus of public funding due to its regenerative and relatively flexible nature. In parallel, the investigation of the microbial community involved in the complex anaerobic degradation process was in the spotlight of microbiome research (Nettmann *et al*., 2008; Bergmann *et al*., 2010; Hanreich *et al*., 2013; Westerholm *et al*., 2015; Güllert *et al*., 2016; Maus *et al*., 2016a; Müller *et al*., 2016; Sun *et al*., 2016). The degradation of organic matter like straw, maize, sugar beet, manure or sewage sludge can be categorized into four major steps: hydrolysis, acidogenesis, acetogenesis and methanogenesis (Cohen *et al*., 1980; Wirth *et al*., 2012). During the initial hydrolysis step, polysaccharides, proteins and fats of the input material are broken down to sugars, amino and fatty acids by fermenting microorganisms (Wirth *et al*., 2012; Güllert *et al*., 2016; Sun *et al*., 2016). In the consecutive acidogenesis, these intermediate degradation products are further transformed to CO_2_, H_2_, volatile fatty acids (VFA) and alcohols. During acetogenesis, acetate, one of the central intermediates, is produced from VFAs or from CO_2_ and H_2_ by secondary fermenters (Wirth *et al*., 2012; Schink and Stams, 2013). During the final methanogenesis step CO_2_, methanol, acetate as well as methylated compounds are reduced to CH_4_ by methanogenic archaea (Thauer *et al*., 2008). While the first three steps are mainly performed by the bacterial community of the biogas plant, methanogenesis is a trait unique to the archaeal domain (Offre *et al*., 2013). The microbial community structure within a biogas plant has been shown to correlate with the feed stock (De Vrieze *et al*., 2015; Theuerl *et al*., 2015), operation temperature (Garcia and Angenent, 2009; Chapleur *et al*., 2015; De Vrieze *et al*., 2015; Luo *et al*., 2016), retention time (Rincón *et al*., 2008; Ho *et al*., 2014; Luo *et al*., 2016) or e.g. ammonia concentration (Schnürer and Nordberg, 2008; Garcia and Angenent, 2009; Theuerl *et al*., 2015). In addition, the microorganisms can flexibly respond to changing environmental conditions to a certain extend (Scherer, 2007; Demirel and Scherer, 2008; Niu *et al*., 2015).

Increased ammonia concentrations are one of the most frequent incidence observed in commercial biogas plants (Chen *et al*., 2008; Yenigün and Demirel, 2013; Lv *et al*., 2014). The microbial community as well as the activity of the methanogenic pathway has been shown to respond to changing ammonia concentrations in various laboratory scale studies (Ahring, 1995; Demirel and Scherer, 2008) as well as in full‐scale reactors (Sundberg *et al*., 2013; Yenigün and Demirel, 2013). Firmicutes and especially Clostridiaceae have been observed to positively correlate with increased ammonia conditions, while Bacteroidales seem to be more abundant under lower ammonium concentrations (De Vrieze *et al*., 2015). On the archaeal side, especially the degradation pathway of acetate was shown to be highly sensitive to the ammonium concentration (Fotidis *et al*., 2014). Under lower concentrations, methane is formed from acetate mostly direct by the acetoclastic pathway performed by Methanosarcinaceae or Methanosaetaceae. With increasing ammonium concentrations, a predominance of syntrophic acetate oxidation coupled to hydrogenotrophic methanogenesis is observed (Zinder and Koch, 1984; Schnürer and Nordberg, 2008). The investigations on the activities of these two alternative methanogenesis pathways, plus the triggers for a change in their abundance, are in the focus of recent studies (Werner *et al*., 2014; Müller *et al*., 2016; Westerholm *et al*., 2016).

Here, we aimed to characterize the changes in the microbial community structure within a full‐scale, mesophilic, anaerobic digester fed with maize silage, dried poultry manure and cow manure over an extended period of time. Regular samples were taken over a period of 587 days. During this sampling procedure, alternating ammonia concentrations were observed, with total ammonium concentrations (NH_4_^+^–N) reaching from 2.7 g l^−1^ to 5.8 g l^−1^ [NH_4_^+^–N]. The microbial composition was correlated to the measured environmental parameters. In addition to the species abundance, metatranscriptomic data were generated for six selected time points to investigate marker gene transcription activity and taxonomic origin aiming to identify major players within the complex community and their reaction to stress introduced by increased ammonia levels.

## Results and discussion

### Reactor conditions during monitoring period

During the monitoring period of 587 days, the mixed substrate utilized was maize silage (67.5% ± 1.4), dried poultry manure (18.6% ± 4.7) and cow manure (13.9% ± 4.3) with on average 1090 tons organic fermenting material per month. The average energy production during the sampling period was 477 kWh h^−1^. While pH remained relatively stable between 7.8 and 8.3, the concentration of ammonium nitrogen [NH_4_^+^–N] in the system increased from 3 g l^−1^ at the start of the investigation to concentrations above 4 g l^−1^ during day 123 to 346 reaching a maximum concentration of 5.8 g l^−1^ at day 226. Thus, the NH_4_^+^–N concentrations, at least during this time period, exceeded the reported ammonia concentration beneficial for acetoclastic methanogenesis which is reported to be below ~3.0 g l^−1^ [NH_4_^+^–N], crucially depending on temperature and pH of the environment (Schnürer and Nordberg, 2008; Fotidis *et al*., 2014). After day 226, NH_4_^+^–N concentrations again decreased relatively constant and reached concentrations around 3.2 g l^−1^ NH_4_^+^–N at the last four sampling time points (from day 492 to 587). The chemical parameters measured and feedstock material for the fermenter during the monitoring period are summarized in Table 1. Feedstock material varied in composition during the monitoring period as a result of substrate availability and market situation.

**Table 1:** Physicochemical parameters of the biogas reactors

Sample ID	Day	MSL (t month^−1^)	DPM (t month^−1^)	CM (t month^−1^)	OLR (kg _oTS_ m^−3^ d^−1^)	CH_4‐calc._ (10^−3^ m^3^ kg_oTS_^−1^ d^−1^)	CH_4‐measured_ (10^−3^ m^3^ kg_oTS_^−1^ d^−1^)	EC (mS cm^−1^)	Ammonia [NH_4_^+^–N g l^−1^]	HRT (days)	VFA/TAC	pH
BGR02	0	231.4	69.4	43.8	4.0	4.10	4.09	35.6	3.1	75.7	0.2	7.9
BGR03	82	218.6	64.7	40.8	3.7	3.86	4.24	34.2	3.0	80.4	0.2	8.0
BGR04	123	222.6	92.7	32.2	4.0	3.94	4.10	40.7	4.4	78.5	0.2	8.0
BGR05	174	237.8	92.3	34.3	4.2	4.02	3.83	41.3	4.4	76.4	0.2	8.2
BGR06	200	224.1	78.1	36.2	4.0	3.87	3.33	45.7	4.9	78.4	0.2	8.1
BGR07	226	229.4	77.3	37.2	4.0	3.87	3.70	50.6	5.8	79.5	0.2	8.0
BGR08	253	223.0	72.9	37.7	4.0	3.90	3.50	48.0	5.1	78.9	0.2	8.3
BGR09	301	221.5	72.4	38.0	3.8	3.83	4.07	47.1	4.9	81.3	0.2	8.1
BGR10	316	236.1	92.8	22.9	4.1	2.64	4.12	44.7	4.7	76.1	0.2	8.1
BGR11	346	211.8	98.7	9.4	4.1	2.61	3.58	44.9	4.1	79.6	0.2	8.1
BGR12	375	242.4	99.6	16.7	4.1	2.66	4.24	38.5	3.7	77.5	0.2	8.0
BGR13	408	203.8	84.1	18.7	3.6	2.38	3.45	36.6	3.4	84.8	0.2	8.0
BGR14	450	232.4	93.5	20.5	4.0	2.60	1.82	39.1	3.8	77.6	0.2	7.9
BGR15	462	227.1	87.3	21.5	4.0	2.61	2.46	41.9	4.0	79.0	0.2	8.1
BGR16	492	250.9	110.3	26.6	4.5	4.44	2.34	34.6	3.0	72.2	0.2	7.9
BGR17	526	275.0	94.9	35.5	4.7	4.47	2.21	31.4	2.9	71.7	0.2	7.8
BGR18	553	261.0	69.1	36.2	4.4	4.33	4.61	29.9	2.7	76.0	0.2	7.8
BGR19	587	222.5	50.8	31.8	3.5	3.52	3.45	35.4	3.2	93.9	0.2	8.0

Day starting from the first sampling, maize silage (MSL), dried poultry manure (DPM), cow manure (CM), organic load weight (OLR), methane amount calculated from input material (CH_4‐calc._), methane amount measured (CH_4‐measured_), electric conductivity (CM), hydraulic retention time in days (HRT), quotient of volatile fatty acids divided by total inorganic carbon (VFA/TAC) and pH. Weight of substrates refers to organic dry mass (oTS). Underlined values for methane were imputed.

**Figure 1:**
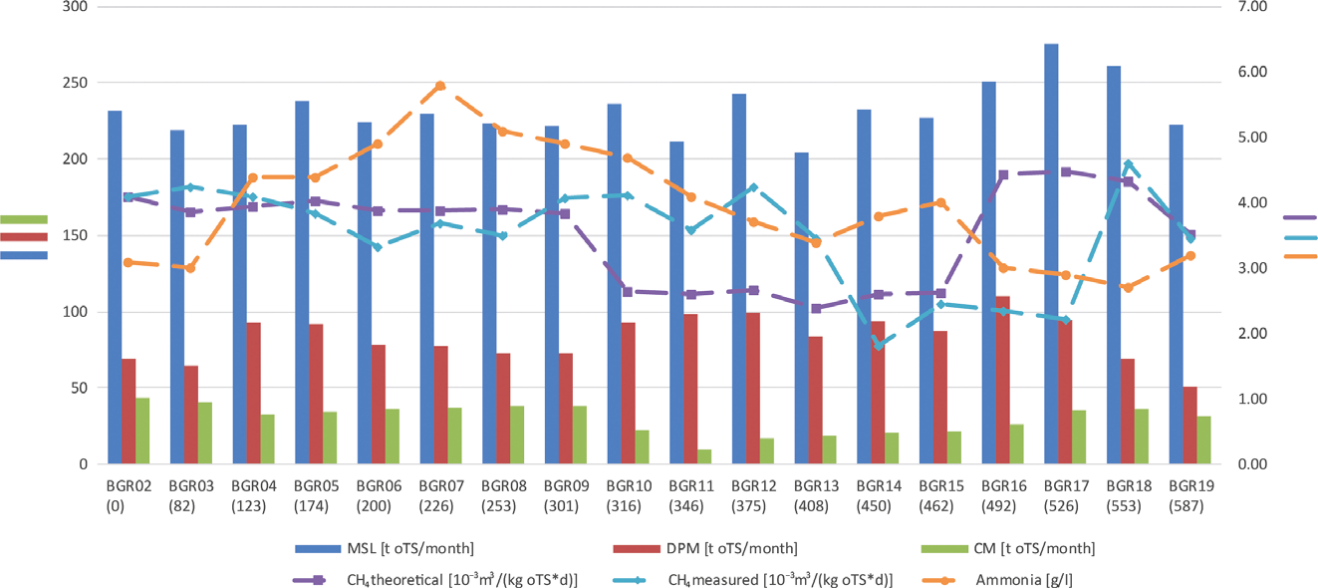
Substrate composition and methane formation (theoretical and measured) during the monitoring period. Dashed lines are applied on the second ordinate.

The energy production of the biogas plant remained relatively stable during the increased ammonia concentrations indicating a still intact anaerobic degradation process and methanogenesis by the microbial community. Methane (CH_4_) production of the reactor material samples remained stable under increased ammonia concentrations as well. CH_4_ production ranged between 1.96 μmol g^−1^ h^−1^ and 2.53 μmol CH_4_ g^−1^ h^−1^ even under ammonia concentrations above 4 g l^−1^ from day 123 to 346 (BGR04‐11). A reduced methanogenesis potential was observed in samples from day 450 to 526 (BGR14‐17). The reduced CH_4_ formation was in accordance with the decreased theoretical CH_4_ formation calculated from incubations of the input material (Table 1, CH_4_ theoretical) and therefore rather reflects the underfeeding of the community than an intoxication by increased ammonia concentrations. Substrate composition, theoretical methane formation, ammonia concentrations and methane production are illustrated in Fig. 1.

### Microbial community rearrangement in response to changing process conditions

**Figure 2:**
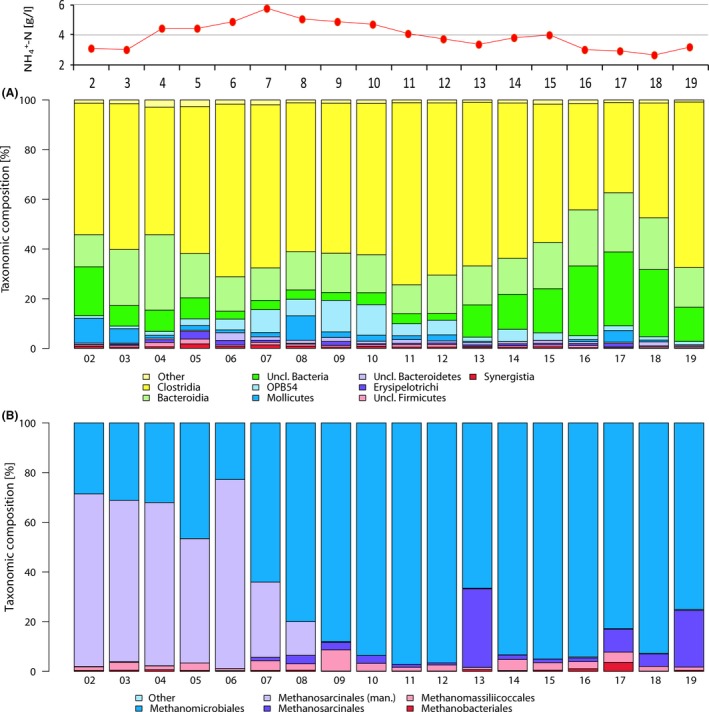
16S rRNA gene‐based community composition for the bacterial (A) and archaeal (B) domain within the studied biogas reactor. Classification was done using the Greengenes database (DeSantis *et al*., 2006) and is shown for the archaea on the order and for the bacteria on the class level. Ammonia concentrations for the samples are shown above the graphs.

The microbial community showed a flexible response to the changing environmental conditions (e.g. NH_4_^+^–N concentration, substrate composition and VFA concentration). The bacterial and archaeal community of the analysed biogas reactor samples (BGR) is illustrated in Fig. 2 on the class level for bacteria and order level for the archaeal part. Initially, the bacterial community of the investigated reactor was dominated by the bacterial phyla Firmicutes, Bacteroidetes and Tenericutes. Firmicutes were present in abundances between 40.3% and 80.0% within the bacterial sequences. The class Clostridia within the order Firmicutes contributed the major part of the sequences within the dataset (see Fig. 2A). The provisional order‐level taxon MBA08 was the most abundant clostridial order. Members of this phylogenetic group were recently observed in thermophilic biogas reactors (Tang *et al*. 2004; Cheon *et al*. 2007; De Vrieze *et al*., 2015), where they largely contribute to cellulose degradation (Lynd *et al*., 2002). Beside this order, Clostridiales also contributed between 4.8% and 20.0% to the clostridial sequences. Clostridiales are known to include a variety of taxa playing key roles during hydrolysis, acidogenesis and acetogenesis as well as syntrophic interactions (Schnürer *et al*., 1996; Hanreich *et al*., 2013; Schink and Stams, 2013; Güllert *et al*., 2016).

With abundances in the dataset between 13.1% and 31.0% during the monitoring period, Bacteroidetes and within them mostly the order Bacteroidales were the second most abundant class within the biogas reactor (11.6%–30.4%). With a percentage up to 10% in the dataset, Acholeplasma was the most abundant class within the phylum Tenericutes, which contributed between 0.5% and 10.4% to the bacterial sequences. Acholeplasma are frequently observed in biogas plants (Krober *et al*., 2009; Solli *et al*., 2014; Bengelsdorf *et al*., 2015) and are possibly involved in polysaccharide degradation and acid production (Freundt *et al*., 1984; Razin, 2006).

Bacteroidetes and Firmicutes consequently contributed the majority of the biogas plant bacteria. They are known as typical colonizers of biogas plants and competitors in the anaerobic degradation of organic material. The ratio of Firmicutes versus Bacteroidetes was in the range of 1.7:1 and 6.1:1, which is comparable to previously described observations in biogas plants (Güllert *et al*., 2016). In other plant biomass degrading habitats, like cow rumen or elephant intestine, significantly lower ratios of Firmicutes versus Bacteroidetes are typically observed as part of the healthy gut flora (Güllert *et al*., 2016). In the above‐mentioned habitats, as well as in the biogas reactor, Firmicutes and Bacteroidetes are involved in the degradation of biomass during the hydrolysis, acidogenesis and acetogenesis (Wirth *et al*., 2012; Güllert *et al*., 2016). During the raising NH_4_^+^–N concentrations in the studied biogas plant, clostridial sequences increased from 52% at the start of the observations up to 65% at the peak observed NH_4_^+^–N concentration (day 226, BGR07). From this point on, a further increase in abundance was observed during ammonium concentration above 4g l^−1^ to maximum abundance of 73% of all bacterial sequences in the dataset at day 346 (BGR11). Meanwhile, bacteria affiliated with the class Bacteroidia contributed between 11.6% and 30.4% of the bacterial sequences and slightly decreased in the overall abundance under the changing ammonia concentrations. The observed positive correlation of Clostridia and increased ammonia levels, which has previously also been reported, indicates a higher tolerance of members of the class Clostridia towards ammonia (Hanreich *et al*., 2013; De Vrieze *et al*., 2015) and their potential contribution to syntrophic acetate oxidation (Westerholm *et al*., 2011; Müller *et al*., 2016; Westerholm *et al*., 2016). Interestingly, the accumulated percentage of unclassified bacteria decreased under high ammonia conditions. Since no further taxonomic information could be found, this shows the need of further isolation and characterization of microorganisms from this divers habitat.

Regarding the archaeal community within the biogas plant, a strong change from a Methanosarcinaceae‐dominated community to a Methanomicrobiaceae‐dominated community was observed during the monitoring timeframe (see Fig. 2B). At the first sampling points, the majority of sequences obtained (represented by purple coloured fraction, 70%) were not classified based on the applied Greengenes database (DeSantis *et al*., 2006) and represented the operational taxonomic unit (OTU) 2. This OTU showed high similarity to *Methanosarcina* strains during manual blast search (Altschul *et al*., 1990). Close relatives were *M. siliciae* and *M. vacuolata*, both with 99% sequence similarity and an *e*‐value of 3 *e* ^−140^. However, the closest similarity with > 99% and an *e*‐value of *e* ^−144^ were observed to an isolate from the very same biogas reactor named *Methanosarcina flavescens*, recently characterized by Kern *et al*. (2016). Methanosarcinales gradually decreased from 70% at day 0 (BGR02) to 3% at day 375 (BGR12) and thereafter slowly increased from 2% at day 450 (BGR14) to 23% in the last sample taken at day 587 (BGR19). Additionally, we observed a single increased abundance of 31% at day 408 (BGR13) which origin could not be explained within the experiment and might potentially originate from sample heterogeneity for once within the habitat at that time point. In addition to these most abundant families, sequences classified as Methanomassiliicoccaceae were observed in all samples with abundance between 0.8 and 8.5%, whereas Methanobacteriales only were present in low abundance and contributed on average 0.6% of the archaeal sequences. Methanomassiliicoccales is a recently identified new order of methanogens (Iino *et al*., 2013; Borrel *et al*., 2016). So far, members of this order have been characterized as H_2_‐dependent methanogens utilizing methanol and methylamines for methane generation (Iino *et al*., 2013) and were reported as part of the archaeal community in other biogas reactors (Stolze *et al*., 2015).

The change in the archaeal community thus appears to be even more pronounced compared to the bacterial community, since we observed the change from a *Methanosarcina*‐dominated to a mainly *Methanoculleus*‐dominated community. Methanosarcinales are known to be the most metabolically versatile group in terms of methanogenesis substrates with members able to utilize methanol, acetate and methylated compounds besides CO_2_ and H_2_ for methanogenesis (Thauer, 1998; De Vrieze *et al*., 2012).

In our effort to correlate the standardized monitoring parameters of the biogas plant (such as feed stock, CH_4_ production, organic load rate, NH_4_^+^–N concentration, hydraulic retention time, pH, VFA/TAC), the changes within the community composition during the monitoring period were further analysed by beta‐diversity analysis.

### Beta‐diversity analysis confirms correlation of Methanomicrobiales and Clostridia with increased ammonia concentration

For a better understanding of the dependency of community changes on process parameters, redundancy analysis (RDA) was applied. OTU counts‐per‐site data generated on the 97% similarity level were normalized and Hellinger transformed. As explanatory variables, the measured parameters ‘Day’, the amount of feed stock of dried poultry manure (DPM), maize silage (MSL) and cow manure (CM), the measured CH_4_ production, the concentration of ammonia nitrogen ‘Ammonia’, the hydraulic retention time (HRT), the VFA to TAC ration and the organic load rate (OLR) were tested in application to the community data. To identify the most relevant explanatory data and prevent variance inflation, variables were submitted to forward selection based on the Akaike information criterion using 1000 permutations (Legendre and Gallagher, 2001; Borcard *et al*., 2011).

**Figure 3:**
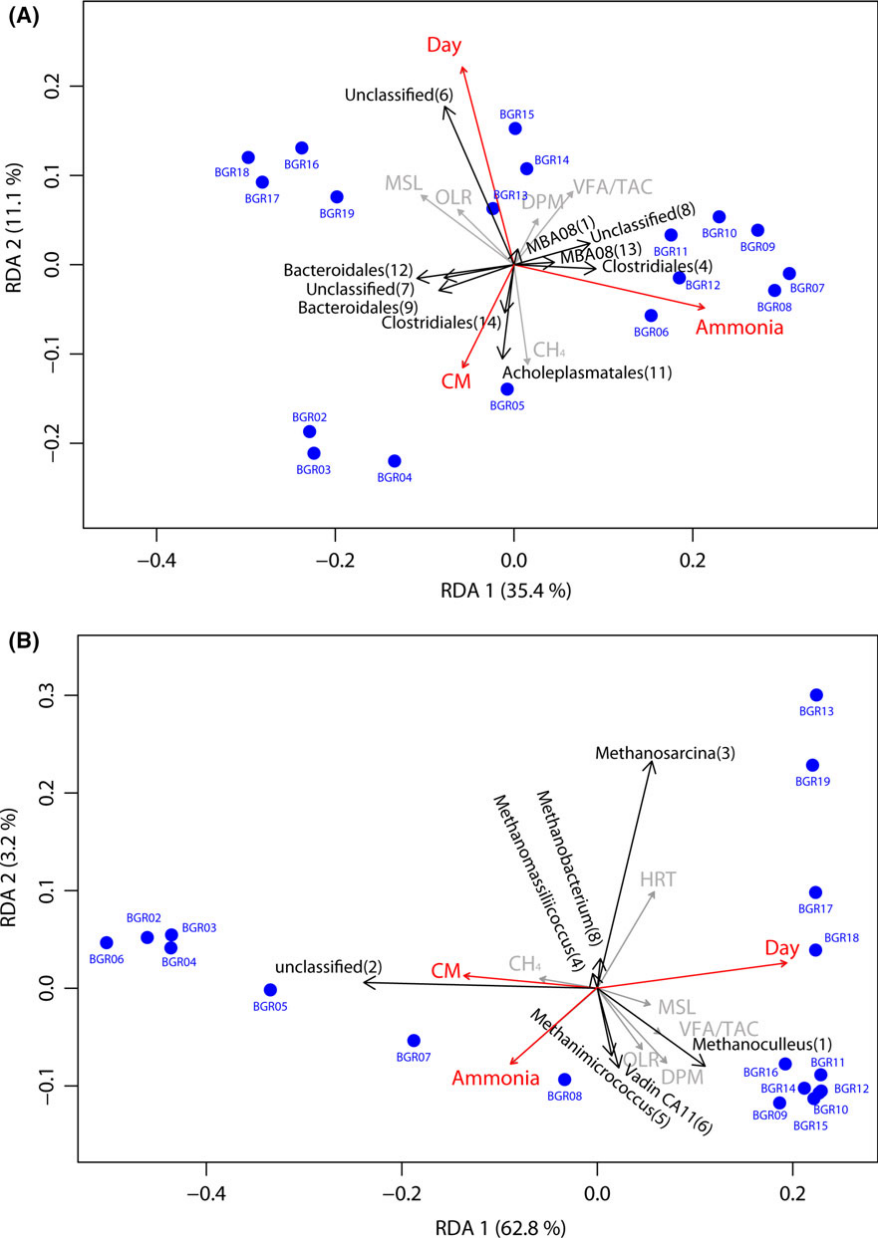
RDA based on Hellinger‐transformed OTU‐count data of the bacterial (A) and archaeal (B) community. Samples are marked by blue dots; environmental parameters contributing to the model are symbolized as red vectors. The OTUs introducing the most variance to the model are shown as black vectors. The explanatory variables are visualized as red vectors. MSL, Maize silage; DPM, dried poultry manure; CM, cow manure; CM, electric conductivity; HRT, hydraulic retention time in days; VFA, volatile fatty acids; TAC, total anorganic carbon.

The final RDA model contained ‘Day’, ‘Ammonia’ and ‘CM’. Redundancy analysis of the archaeal and bacterial datasets showed a changing community composition during the monitoring period (Fig. 3A and B). The applied model was tested for significance using the command anova with 1000 permutations. Results showed high significance for the archaeal (*F*_3,14_ = 12.074; *P* < 0.001) and bacterial (*F*_3,14_ = 6.793; *P* < 0.001) models. The overall variance explained by the first two axis of the RDA was 66.2% for the archaeal and 50.6% for the bacterial dataset. Ammonia concentration and the progressing monitoring time as ‘Day’ were the two variables explaining most of the variance within the RDAs.

In the visualization of the RDA gained from the bacterial community composition, a clear separation of the samples can be observed (Fig. 3A). The vectors relating to the 10 OTUs with the most contribution to the observed variation were extracted from the RDA model. Vectors representing OTU11 (*Acholeplasma*) and OTU14 (Clostridiales) were characteristic for the early biogas plant samples (BGR02‐05; day 0‐day 174). Reactor samples from time points with an increased ammonia concentration (BGR07‐12; day 226‐day 375) clustered together and were better characterized by OTU8 (OPB54), OTU3 (MBA08) and OTU4 (*Caldicoprobacter*). All of these OTUs belonged to the phylum Firmicutes, OTU3 and OTU4 were assigned to the class *Clostridia,* a class known to harbour a variety of syntrophic organisms (Schnürer *et al*., 1996; Schink and Stams, 2013). Representatives of the class OPB54 were initially found in sequences from hot springs (Hugenholtz *et al*., 1998) and have been recently observed in biogas samples, especially abundant under increased load rate and reactor acidification (Roske *et al*., 2014). A first isolate of this candidate taxon, *Hydrogenispora ethanolica*, was obtained from a mesophilic anaerobic sludge reactor, treating herbicide waste. It was characterized as a strictly anaerobic, spore‐forming, hydrogen‐producing bacterium utilizing various saccharides (Liu *et al*., 2014). Strong positive correlation (Pearson correlation > 0.7; *P* < 0.01) was observed for the above‐mentioned OTUs with the factor ammonia. Moderate negative correlation with increased ammonia concentrations was observed for the OTU12 (uncl. Bacteroidia, Pearson < −0.55; *P* < 0.02), OTU9 (BF311, Pearson < −0.71 *P* < 0.001) and OTU7 (uncl. Bacteria, Pearson < −0.70; *P* < 0.002), two of which belonging to the order Bacteroidales. Increased ammonia concentrations are therefore most likely contributing to the dominance of Clostridiales over Bacteroidales in this biogas plant. A similar observation was recently reported for laboratory scale reactors (Hanreich *et al*., 2013). The biogas plant samples from the later sampling points were more characterized by the presence of OTU6, which was classified as uncultivated Bacteria. Manual blast search using the NCBI non‐redundant nucleotide database (version 70) showed high similarity to sequences isolated from anaerobic digesters (Kampmann *et al*., 2012); however, no further classification and functional prediction were available for this taxon. This further underlines the need for continued isolation and characterization of microorganisms to increase our understanding on the processes in complex environments (Kern *et al*., 2016; Maus *et al*., 2016a,b).

In the visualization of the RDA gained from the archaeal community composition, similar separation of the samples can be observed (Fig. 3B). The vectors relating to the seven OTUs with the most contribution to the observed variation were extracted from the RDA model. In the archaeal dataset, OTU2 was highly characteristic for the early monitoring time points. This was already observed in the taxonomy composition bar plots, as OTU2 was identified as most likely *Methanosarcina flavescence* and highly abundant in the early community (BGR02‐05; day 0 to day 174). OTU1, which was classified as *Methanoculleus*, as well as OTU5 classified as *Methanomicrococcus* and OTU6 classified as the closely related to the isolate Vadin CA 11 (Methanomassiliicoccaceae) (Godon *et al*., 1997) were characteristic for biogas samples from the middle of the monitoring period characterized by increased ammonia concentrations (BGR07‐15; day 226–day 462). In the later biogas plant samples, with again lower ammonia content, OTU3 assigned to the genus *Methanosarcina* became more abundant and characteristic for the archaeal community.

### Transcript abundance of genes encoding for key enzymes support the proposed reduction of acetoclastic methanogenesis under increased ammonia concentration

Based on the observation of the archaeal community and bacterial community by 16S rRNA gene‐based analysis, we assume an acetoclastic pathway during the early stages of the monitoring period and dominance of hydrogenotrophic methanogenesis under increased ammonia conditions. To further validate this hypothesis, metatranscriptomic datasets were generated for six selected sampling points (days 82, 174, 226, 253, 553 and 587).

The taxonomic composition in the metatranscriptome as analysed using the software kaiju (Menzel *et al*., 2015) showed a different community compared to the observations from the 16S rRNA gene‐based community composition (Fig. S1). Based on the analysis of the metatranscriptomic reads, archaea contributed between 19.5% and 32.6% to the transcriptional activity of the overall community.

The archaeal community as observed by the metatranscriptome was dominated at all sampling points by Methanomicrobiales (between 82% and 93% of all reads annotated as archaea). Methanosarcinales were in the range between 13% and 3%. Smaller amounts of Methanobacteriales and Methanomassiliicoccales were additionally observed. Similar differences to the 16S rRNA gene‐based analysis were observed in the bacterial domain. While Clostridia and Bacteroidia remained highly abundant classes, their abundance dropped from 66% to 49% of the overall dataset. Therefore, Bacilli, γ‐ and δ‐Proteobacteria as well as Actinobacteria were also found in higher abundance compared to the 16S rRNA gene‐based community analysis. These differences between the two methods were expected, and result from different databases used for analysis, different overall bioinformatic procedures, but mostly from the difference between DNA‐ and RNA‐based approaches. While the 16S rRNA gene‐based analysis using environmental DNA allows the identification of present microbial organisms, the RNA‐based analysis by metatranscriptomic shotgun sequencing is allowing the analysis of actively transcribed genes and a prediction of metabolic activity. Therefore, the community composition as observed by the taxonomic annotation of the metatranscriptomic reads rather identifies active organisms within the environment under the given conditions (Zakrzewski *et al*., 2012; De Vrieze *et al*., 2016).

**Figure 4:**
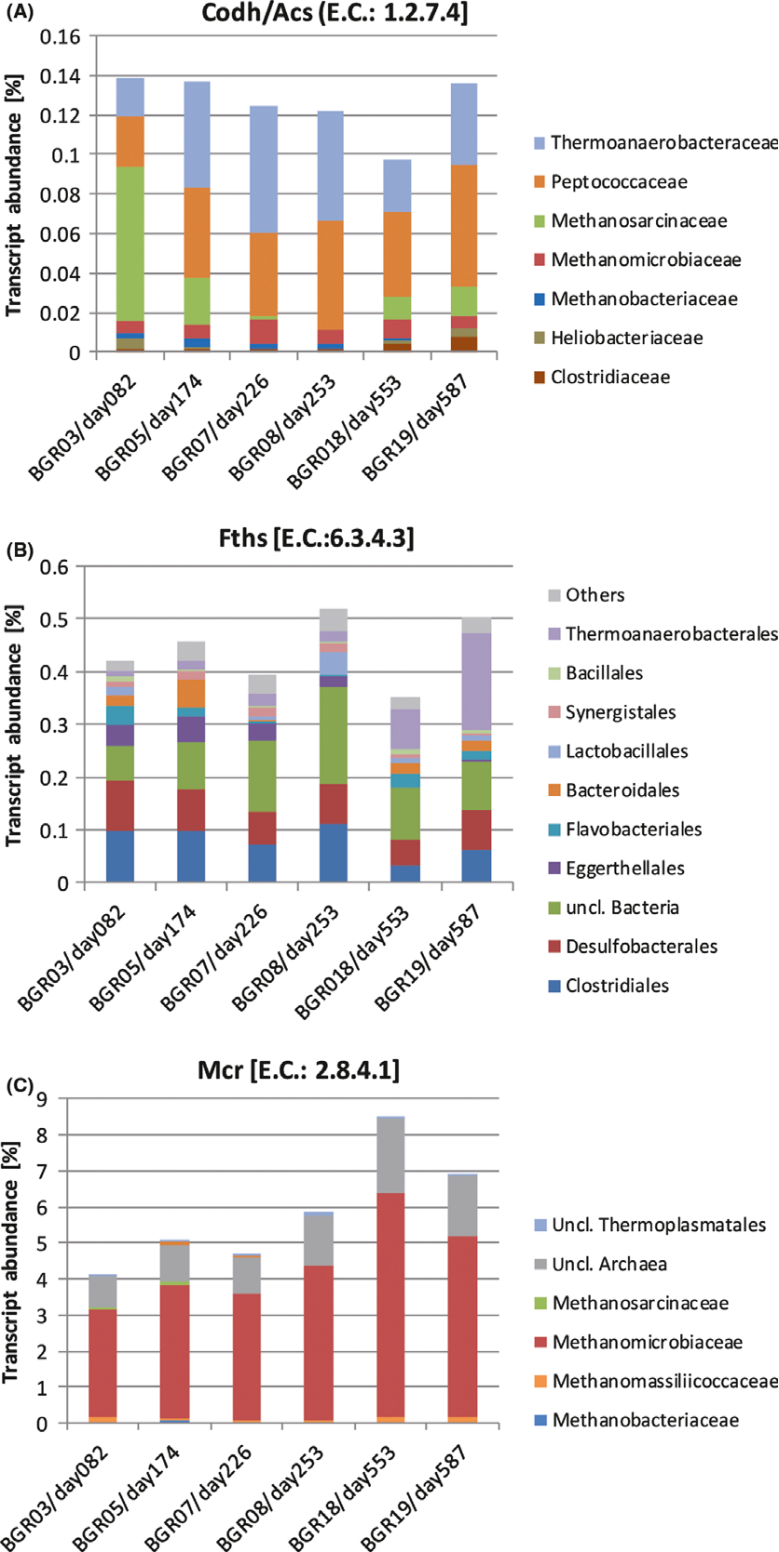
Transcriptional abundance of the genes encoding for the enzymes Codh/Acs (A), Fthfs (B) and Mcr (C). The taxonomic composition of the actively transcribing community was obtained by the annotation of the contigs harbouring the investigated marker gene.

To verify the presence and annotation of genes involved in the degradation and turnover of organic material, we checked the completeness of the central carbon metabolism (KEGG map 001200) based on E.C. numbers present in our dataset using the KEGG mapper online tool (see Fig. S) (Kanehisa and Goto, 2000; Kanehisa *et al*., 2017). Within the context of biogas production, of special interest was the transcription genes for enzymes involved in the methanogenic pathway. To specifically quantify acetoclastic methanogenesis, transcripts encoding the carbon monoxide dehydrogenase/acetyl‐coenzyme A synthase (Codh/Acs, E.C. 1.2.99.2) were investigated in regards of their taxonomic origin and abundance within the dataset. Since this gene is present as well in bacteria capable of the Wood–Ljungdahl pathway, taxonomic classification against the NCBI non‐redundant nucleotide database release 70 was performed to differentiate between archaeal and bacterial transcripts. Overall, between 0.140% and 0.097% of all transcripts were annotated to code for Codh/Acs. While the majority (0.08%) of the 0.140% of transcripts from day 82 (BGR03) were annotated as coding for Codh/Acs and taxonomically classified as originating from Methanosarcinaceae, the transcriptional activity of this taxon decreased below the detection limit in the dataset of the metatranscriptomic sample taken at day 253 (BGR08) (see Fig. 4A). After the strong initial reduction, a partial regeneration of the abundance form transcripts of this taxonomic group was observed in the latter two metatranscriptomic samples (0.01% at day 553 (BGR18) and 0.02% at day 587 (BGR19)), most likely indicating a possible regeneration of the Methanosarcinaceae community and at least partial reconstitution of acetoclastic methanogenesis. Reduction and reoccurrence of transcripts originating from Methanosarcinaceae were in accordance with the results obtained by 16S rRNA gene sequencing and appear to be negatively linked to ammonia concentration. Similar observations have been reported on the proteomic level but not yet on a transcriptional level (Theuerl *et al*., 2015). To our knowledge, a direct transcriptional response on the marker gene level of a community within a commercial biogas plant to changing ammonia concentration over an extended period is presented here for the first time.

During the increased ammonia concentration, transcription of bacterial genes for the Codh/Acs affiliated with Thermoanaerobacteraceae and Peptococcaceae increased. As mentioned above, the carbon monoxide dehydrogenase/acetyl‐CoA synthase is a key enzyme within the Wood–Ljungdahl pathway and in syntrophic acetate oxidation (SAO). SAO coupled to hydrogenotrophic methanogenesis is the alternative pathway for acetate utilization and methanogenesis, especially abundant under increased ammonia conditions (Müller *et al*., 2016; Westerholm *et al*., 2016). Beside the Codh/Acs, the gene coding for formyl‐tetrahydrofolate synthetase (Fthfs, E.C. 6.3.4.3) has been applied recently as a marker gene in studies investigating SAO (Müller *et al*., 2016). Taxonomic annotation of the transcripts from the Fthfs showed higher diversity compared to the transcripts for Codh/Acs and provided evidence for an increased activity of Clostridia in SAO during high NH_4_^+^‐N conditions (Fig. 4B). Clostridia, including the classes Clostridiales, Thermoanaerobacterales and Haloanaerobacteriales, were the most active class in terms of the transcription for the *fthfs* genes. In particular, Thermoanaerobacterales were increased in abundance during and after higher ammonia concentrations (BGR07‐BGR19), contributing up to 37% to the overall Fthfs transcripts. This family includes typical syntrophic organisms such as *Syntrophaceticus schinkii*, a typical syntrophic acetate‐oxidizing organism found in anaerobic fermenters (Westerholm *et al*., 2010; Mosbaek *et al*., 2016). Beside Clostridia, Deltaproteobacteria, Bacteroidia, Flavobacteria and Bacilli were identified as transcriptionally active classes regarding the *fthfs* genes. In addition, a high percentage between 34% and 35% of unclassified Bacteria was observed during increased ammonia concentrations, indicating that still a large number of syntrophic organisms are uncharacterized.

An overall increase in bacterial transcription of genes encoding for Codh/Acs and Fthfs, as well as a reduction of methanosarcinaceal transcripts encoding for Codh/Acs, provides strong evidence for a redirection of the acetate degradation flow from direct acetoclastic methanogenesis to syntrophic acetate oxidation coupled to hydrogenotrophic methanogenesis caused by increased ammonia concentration. Methanomicrobiales show a low percentage of the transcripts encoding for Codh/Acs of 0.006 to 0.012% within the dataset, compared to up to 6.2% of the transcripts coding for the Methyl‐Coenzyme M reductase (Fig. 4A and C). These observations most likely result from the fact that Methanomicrobiales utilize their CODH/ACS for acetate activation and biosynthesis of new cellular biomass. Methanosarcinales however, when metabolizing acetate additionally, depend on this enzyme for their energy metabolism (acetoclastic methanogenesis). Therefore, transcription levels in Methanosarcinales were up to 14‐fold higher compared to Methanomicrobiales during possible active acetoclastic methanogenesis (BGR03).

To additionally gain information on the overall proportions between acetoclastic and hydrogenotrophic methanogenesis, the transcriptional activity and taxonomic origin of the Methyl‐Coenzyme M reductase (Mcr, E.C. 2.8.4.1) was further analysed (Fig. 4C). As observed in the transcriptional activity, hydrogenotrophic methanogenesis appeared to be the dominating pathway since the majority of the reads coding for the Mcr (72%–75%) were affiliated with the Methanomicrobiaceae, a strictly hydrogenotrophic family. Methanosarcinaceae were more abundant in the early reactor samples with a presence of 2% in the sequences encoding for the Mcr while decreasing in the later once down to no detectable reads at day 253 (BGR08). The high percentage of transcripts encoding for Mcr of up to 8.5% at day 553 (BGR18) of all annotated reads reflects the importance of this enzyme as a key enzyme for methanogenesis. Consistent with this finding BGR18 also showed the highest CH_4_ production rate within the monitoring period (Table 1, Fig. 1). A high abundance of genes coding for enzymes involved in methanogenesis, especially for the Mcr, was already reported in other studies on biogas reactors (Hanreich *et al*., 2013; Heyer *et al*., 2013, 2015). Therefore, our results are in accordance with previous observation.

Noteworthy, a discrepancy was observed between the abundance of Methanosarcinaceae in the 16S rRNA gene‐based analysis of the community as well as their contribution to the acetate degradation as observed by their transcriptional activity of the genes coding for the Codh/Acs and their low contribution to methanogenesis as ascertained by the low number of transcripts for the marker enzyme Mcr. Such effects have already been observed for other environments and reflects the often overseen crucial difference between abundance and activity within a microbial community (Helbling *et al*., 2012; Chen *et al*., 2015; Coordinators, 2016; De Vrieze *et al*., 2016). To further elucidate the actual activity of the alternative methanogenic pathways (syntrophic acetate oxidation coupled to hydrogenotrophic methanogenesis or direct acetoclastic methanogenesis), metaproteomic (Heyer *et al*., 2015; Theuerl *et al*., 2015) or isotope labelling experiments for the identification of the active community involved in the degradation process (Li *et al*., 2009; Hyatt *et al*., 2012; Lünsmann *et al*., 2016; Westerholm *et al*., 2016) are required in future work.

### Transcriptional activity of metagenomic bins over time

In a recent publication, Güllert *et al*. (2016) investigated the cellulolytic potential of the very same biogas reactor by an in depth metagenomic study. Within this study, several high‐quality metagenomic bins were generated and taxonomically annotated in detail for one selected time point (BGR03). Building up on these results, we investigated the transcriptional activity of 20 high‐quality bins (completeness > 80%, contamination < 10% and heterogeneity (of the contamination) < 50%) at five additional time points. Therefore, a subset of 29.6 million metatranscriptomic reads from each sampling point were mapped on these bins to estimate their overall transcriptional activity at the sampled time points. The genomic bins showed different coverages in the range between 33.9‐fold and 0.04‐fold (Table S1), indicating different levels of transcriptional activity. The most active bins with a coverage above 10 were pb80 (Firmicutes), pb3 (*Lachnoclostridium phytofermentans*), pb85 (*Methanosarcia barkeri*), pb31 (*Treponema*) and pb215 (*Ruminiclostridium*). Firmicutes (pb80) and within this phylum especially Clostridia (pb3, pb215) again were found to be highly active within the community and showed a high activity of genes coding for enzymes involved in the degradation of plant material such as cellulosome‐anchoring protein, cellulase, xylanase and endolyase (Table S2). The eight bins with the highest variance over the monitoring period are illustrated in Fig. S3, showing that transcriptional activity of these organisms in fact differed during the observation period. The analysis of the open reading frames (ORFs) showed, beside tRNA regions, transcription factors and ribosomal RNA, that genes coding for enzymes involved in the degradation of plant material (e.g. cellulosome‐anchoring protein, trehalose import ATP‐binding protein, pullulanase, carbohydrate binding domain), cell adherence (e.g. biofilm formation stimulator VEG) and methanogenesis (e.g. Methyl CoM reductase and Codh/Acs subunits) were highly active in the analysed bins (Table S2). In two cases (pb121 and pb80), several phage‐related ORFs (e.g. integrase core domain, transposase, minor tail protein) were observed to be highly active, showing that viral infection appears to be a influencing and potentially shaping the microbial community, as previously shown in other environments (Fuhrman and Schwalbach, 2003; Weitz and Wilhelm, 2012; Dutilh *et al*., 2014). In summary, the high transcriptional activity of some of the investigated bins points to an important function of the corresponding microorganisms in the microbial community of the studied biogas reactor. In particular, enzymes involved in degradation of plant material and methanogenesis were highly transcribed, highlighting the relevance of these organisms for the anaerobic digestion process of biomass.

## Summary

The methanosarcinal‐dominated community changed in co‐occurrence with increasing ammonia concentrations to a *Methanoculleus*‐dominated community. Simultaneously, we observed a reduction in the transcriptional activity of genes encoding for the archaeal Codh/Acs as a key enzyme for the acetoclastic methanogenesis. Within the bacterial community, a positive correlation between mostly clostridial taxa and increasing ammonia concentrations was observed. Additionally, an increase in bacterial Codh/Acs and Fthfs transcripts within the dataset was observed. Overall, our data indicate a change in the primary acetate pathway from acetoclastic methanogenesis (by Methanosarcinales) to syntrophic bacterial acetate oxidation coupled to hydrogenotrophic methanogenesis (by Clostridia and Methanomicrobiales) by the restructuring the community composition and pathway redirection. After the reconstitution of moderate ammonia levels, first signs of recovery were observed for the acetoclastic pathway on the transcript level and on the 16S rRNA gene abundance level. Within this study, we succeeded in the demonstration of the flexible response of the microbial community in a commercial scale biogas plant to changing ammonia and feed stock conditions over time. The integration of transcriptomic data and the long‐term character of our investigation allow for the first time a deeper insight into the full‐scale biogas reactor microbiome and its functionality.

## Experimental procedures

### Reactor conditions and sampling

A total of 18 reactor samples were taken monthly starting from March 2013 until October 2014 of a commercial mesophilic anaerobic biogas reactor located near cologne. Primary feeding consisted of maize silage, cattle manure and poultry dry manure. The reactor operated at 40°C. Average power production during the sampling period was 477 kWh h^−1^. Important physicochemical parameters are summarized in Table 1. Samples were collected in sealed plastic bottles, transported under cooled conditions and stored in the laboratory at −80°C until nucleic acid extraction. Methane production of the biogas reactor samples was measured as previously described (Refai *et al*., 2014). Shortly, 20 g of biogas reactor material was transferred into 120 ml serum flasks under anaerobic conditions, sealed with a rubber stopper and purged with N2/CO2 (50/50%, 1 atm) for 10 min. After 24 h incubation at 40°C and 200 r.p.m., the volume of biogas produced was measured using a gastight glass syringe. The CH4 content of the biogas produced was measured via gas chromatography. Briefly, 20 μl of biogas was used for the measurement of the methane content using a Clarus^®^ 480 instrument (PerkinElmer, Waltham, MA, USA) connected to a flame ionization detector and a Rascon FFAP column (25 m, 0.25 micron, PerkinElmer). The temperatures of injector, detector and column oven were 150, 250 and 120°C respectively. A gas mixture of 10% CH_4_ and 90% argon (Air Liquid, Düsseldorf, Germany) was used as standard. Methane production was recalculated referring to cubic metres produced per kg organic dried substrate per day $[m_{{\mathrm{CH}}_{4}}^{3}/\left({\text{kg}}_{\mathrm{oTS}}\times d\right)$.

Missing methane production values (BGR15 and BGR16) was imputed using the method ‘norm.predict’ of the R package mice (Little and Rubin, 2002; Buuren and Groothuis‐Oudshoorn, 2011).

Substrate composition, theoretical methane formation, ammonia concentrations and methane production are illustrated in Fig. 1 and summarized in Table 1.

### Nucleic acid extraction

#### DNA

Isolation of DNA from the samples was conducted using a modified CTAB‐based method modified after Weiland‐Bräuer (Weiland *et al*., 2010). Shortly, 1.5 g of frozen sample material was mechanically disrupted using a Dismembrator U instrument (Sartorius AG, Göttingen, Germany). 2.7 ml DNA extraction buffer with 5% CTAB was added to 1 g of homogenized material. Extracted DNA was highly contaminated by humic acids indicated by brownish to yellow colour. Contamination was removed by using the FastDNA™ SPIN Kit for Soil (MP Biomedicals, Solon, OH) excluding the initial lysis steps. The purity of DNA was analysed using a Nanodrop ND‐2000 instrument (PEQLAB Biotechnologie GmbH, Erlangen, Germany).

#### RNA

RNA was extracted from ~500 mg frozen reactor sample. Therefore, samples were frozen in liquid N_2_ and homogenized using an dismembrator instrument (Sartorius AG, Göttingen, Germany) with 2000 r.p.m. for 5 min. Samples were transferred into 2.5 ml Isoyl‐RNA Lysis reagent (5 Prime GmbH, Hilden, Germany) and RNA was extracted applying the Direct‐zol RNA Kit (Zymo Research, Freiburg, Germany). Protocol for RNA purification included on‐column DNase I treatment (30U, Zymo Research, Freiburg, Germany) according to manufacturer instruction. Extracted RNA was stored at −80°C until further preparation.

### 16S rRNA gene microbial community analysis

#### PCR and amplicon sequencing

Primers applied for the amplification of the amplification of the bacterial and archaeal 16S rRNA gene fragments are listed in Table 2 and were previously tested for applicability to monitor the microbial community in biogas environments (Fischer *et al*., 2016). Extracted DNA was adjusted to a concentration of 20 ng μl^−1^ and applied in the amplification reaction. PCR conditions, purification and pooling were performed as recently described (Mensch *et al*., 2016) with a modified annealing temperature for archaeal primers at 56°C. Final library pool for sequencing was combined from the eluates and contained 100 ng of DNA.

**Table 2:** Sequences of the primers used for 16S rRNA gene amplicon sequencing of the bacterial and archaeal domain

	Primer name	Sequence of the primers
Archaea	Fwd Ar0787	AATGATACGGCGACCACCGAGATCTACACACACTCTTTCCCTACACGACGCTCTTCCGATCT **ATTAGATACCCSBGTAGTCC**
	Rev Ar1059	CAAGCAGAAGACGGCATACGAGATGTGACTGGAGTTCAGACGTGTGCTCTTCCGATCT **GCCATGCACCWCCTCT**
Bacteria	Fwd Ba0027	AATGATACGGCGACCACCGAGATCTACACTATGGTAATTGT **AGAGTTTGATCCTGGCTCAG**
	Rev Ba0338	CAAGCAGAAGACGGCATACGAGATAGTCAGTCAGCC **TGCTGCCTCCCGTAGGAGT**

Primer sequence consisted of an initial standardized Illumina adapter (regular), followed by an eight nucleotide barcode (X's) and a primer sequence (bold). Additionally, primers for the 16S analysis contained a linker sequence for the sequencing reaction on the flow cell (underlined).

The amplicon library sequencing was performed on a MiSeq instrument. Library therefore was prepared according to the manufacturer's instructions and sequenced using the v3 chemistry with 2 × 300 bp paired end.

#### Trimming of amplicon reads

Reads generated with amplicon sequencing were trimmed using the trimmomatic software version 0.33 (Bolger *et al*., 2014). Shortly reads were analysed with a sliding window of 4 bp. Regions were trimmed if the average Phred score (Ewing and Green, 1998; Ewing *et al*., 1998) within the window was below 30. Additional reads were analysed for remaining Illumina adapters and primer sequences. Trimmed reads were kept within the dataset if the forward and reverse read both survived the quality trimming and were longer than 36 bp.

#### Mothur

Quality trimmed sequences were analysed using mothur software, version 1.35.1 (Schloss *et al*., 2009). The analysis of the reads was performed as described recently (Li and Durbin, 2009). Raw reads were concatenated to 2 569 651 bacterial and 1 409 683 archaeal contiguous sequences (contigs) using the command *make.contig*. Contigs were filtered for ambiguous bases, homopolymers longer than eight bases or sequences longer than 552 bases using the command *screen.seqs*. The remaining 1 820 026 bacterial and 1 170 930 archaeal contigs were screened for redundant sequences using the command *unique.seqs* and clustered within 365 485 bacterial and 116 181 archaeal unique sequences. The sequences were consecutively aligned to a modified version of the SILVA database release 102(Pruesse *et al*., 2007) containing only the hypervariable regions V1 and V2 for the bacterial and V5 and V6 for the archaeal sequences using the command align.seqs. Sequences not aligning in the expected region were removed from the dataset using the command screen.seqs. The alignment was further optimized by removing gap‐only columns with the command filter.seqs. The alignment contained 1 361 951 bacterial sequences (292 896 unique) and 1 076 985 archaeal sequences (93 168 unique). Rare and closely related sequences were clustered using the commands unique.seqs and precluster.seqs. The latter was used to cluster sequences with up to three positional differences compared to larger sequence clusters together. Chimeric sequences were removed using the implemented software uchime (Edgar *et al*., 2011)via the command *chimera.uchime*, followed by *remove.seqs* leaving 995 576 bacterial sequences (50 215 unique) and 1 041 591 archaeal sequences (12 689 unique) in the dataset. The classification of the sequences was performed using the Greengenes database (Version 13_05_99) (DeSantis *et al*., 2006) with a bootstrap threshold of 80%. Sequences belonging to the chloroplasts or mitochondria were removed from the dataset. Operational taxonomic units (OTUs) were formed using the average neighbour clustering method with the command *cluster.split*. Parallelization of this step was done taking the taxonomic classification on the order level into account. A sample‐by‐OUT table on the 97% level, containing 20 328 bacterial and 266 archaeal OTUs, was generated using the command *make.shared*. OTUs were classified taxonomically using the modified Greengenes database mentioned above and the command *classify.otu*. The datasets for the individual samples were subsampled to 28,000 bacterial and 11,000 archaeal counts per sample for subsequent bioinformatic analysis.

#### 16S amplicon analysis in R

Analysis and visualization were carried out using the r version 3.2.4 (R Core Team, 2015) and the packages vegan (Oksanen *et al*., 2015), Hmisc (Harrell and Dupont, 2008), dichromat (Lumley, 2013) and scales (Wickham, 2012). After removal of low abundant OTUs (< 0.2% within the dataset), absolute abundance of OTUs generated on the 97% similarity level was transformed using Hellinger transformation (Legendre and Gallagher, 2001) by application of Equation (1).

(1) $$y_{\mathrm{ij}}^{\prime }=\sqrt{\frac{y_{\mathrm{ij}}}{y_{j+}}}$$

where *y*_*ij*_ is the abundance of OTU *i* in sample *j* and *y*_*j*+_ is the sum of all OTUs in sample *j*. $y_{\mathrm{ij}}^{\prime }$ is transformed abundance of OTU *i* in sample *j*.

Redundancy analysis (RDA) was performed to explore the change in OTU composition with explanatory variables ammonia concentration (‘Ammonia’), over time (‘Day’) and the percentage of cow manure (‘CM’) to the overall feed stock. Response data were the Hellinger‐transformed count data. Analysis of variance (ANOVA) was applied with 1000 permutations to test if effects as well as their interaction were statistically significant (Borcard *et al*., 2011).

### Metatranscriptomic analysis

#### Sequencing of metatranscriptome

RNA was prepared for sequencing using the TruSeq reagent Kit, including degradation of ribosomal RNA according to manufacturer's protocol using the RiboZero (Illunina, CA, San Diego). Sequencing of RNA from the reactor units was performed on a NextSeq Instrument (Illunina) using the NextSeq 500 Mid Output Kit for 300 cycles according to the manufacturers protocol.

### Assembly, annotation and transcriptional activity analysis

Raw sequences (345*10^6^ reads) were trimmed using the software trimgalore v 0.3.7 (Krüger, 2012) in paired end mode including adapter trimming, quality check for a Phred value above 30 (Ewing and Green, 1998; Ewing *et al*., 1998) and a minimum sequence length of 20 bp leaving 330 × 10^6^ high‐quality reads with an average read length of 122 bp in the data set. Beside the metatranscriptomic raw reads, metagenomic sequences generated from the same biogas reactor and recently analysed in Güllert *et al*. (Güllert *et al*., 2016) were integrated in the assembly for further improvement. Quality of remaining reads was assessed with fastqc (Andrews, 2010). megahit v 1.0.2 (Li *et al*., 2015) was used for the hybrid assembly of all reads generated for the different time points as well as from the corresponding metagenome using a succession of k‐mer values of 21, 41, 61, 81, 91 and 99. Contigs with a length below 500 bp or coverage under 6 were omitted from the assembly. 248 492 contigs were assembled during the process containing 466 013 840 bp. The longest contig contained 189 023 bp, N50 value of the assembly was 2893 bp. Backmapping of the individual reads was performed with bbmap v35.37 (Bushnell, 2014) to calculate read recruitment and contig coverage for the individual sample datasets. Recruitment rate was calculated to be 54%. Samtools was used for the sorting of the mapped reads. RNAhmm3 (Lagesen *et al*., 2007) was used for removal of ribosomal RNA sequences. metaprodigal v 2.6.2 (Hyatt *et al*., 2012) was used for the gene prediction. Threshold for predicted genes was set to a minimum length of 80 amino acids. Predicted genes were assigned to their potential function using interproscan v5.14‐53.0 (Quevillon *et al*., 2005) for searching against the Pfam‐A version 28.0 (Bateman *et al*., 2004; Finn *et al*., 2014), tigrfam version 15.0 (Haft *et al*., 2003) and superfamily (version 1.75) (Madera *et al*., 2004; Wilson *et al*., 2006) databases. HTSeq (Anders *et al*., 2015) was used for calculation of read recruitment on predicted genes. megablast (Altschul *et al*., 1990, 1997) was used for taxonomic annotation of the contigs using the NCBI nucleotide databases release 70 (Sayers *et al*., 2009). After initial annotation, TAMER (Jiang *et al*., 2012) was used to resolve conflicts with multiple best reference hits. Results of the different modules were transformed to a PostgreSQL database using an in‐house developed R script.

Transcriptional activity of the genes was analysed by backmapping and counting of the sequencing reads on the respective ORF. Taxonomic annotation of the contigs containing the respective ORFs was performed against the NCBI nucleotide database (release 70) (Sayers *et al*., 2009; Coordinators, 2016; O'Leary *et al*., 2016; Coordinators, 2017).

Genes of interest were extracted from the dataset using their respective enzyme commission reference number (E.C. number). As an additional filter step, reads not annotated as bacterial or archaeal were removed from the dataset, since the focus was set on the microbial community. The E.C. read counts of the respective genes were normalized to absolute abundance of functionally annotated genes within their corresponding dataset.

### Analysis of microbial community composition based on individual metatranscriptomic shotgun reads

To assess the taxonomic affiliation of the transcriptionally active organisms, metatranscriptomic shotgun reads were classified using kaiju 1.6.2 (Menzel *et al*., 2015). Taxonomic classification of the reads was conducted using the proGenomes database (Mende *et al*., 2017) as reference which encompasses 25.038 consistently annotated bacterial and archaeal genomes. Kaiju was run in Greedy mode allowing five substitutions and filtering of low‐complexity regions as well as an *e*‐value cut‐off of 0.05 was applied.

### Transcriptional activity of metagenomic bins

The bioinformatical analysis of the bins included the subsampling of the individual samples to 29.6 million reads. These were mapped on the contigs of the 20 high‐quality bins from Güllert *et al*. (2016) using BWA version 0.7.17 (Li and Durbin, 2009) and samtools version 1.1 (Li *et al*., 2009). For functional analysis, ORF prediction and annotation of the bins were done using prokka version 1.12 with standard settings (Seemann, 2014), which implements the tools bioperl (Stajich *et al*., 2002), GNU Parallel (Tange, 2011), BLAST+ (Camacho *et al*., 2009), HMMER3 (Finn *et al*., 2011), Aragon (Laslett and Canback, 2004), Prodigal (Hyatt *et al*., 2010) as well as the databases hamap version 1.1 (Lima *et al*., 2008; Pedruzzi *et al*., 2012) and pfam version 31.0 (Bateman *et al*., 2004; Finn *et al*., 2014).

## Conflict of interest

None declared.

## Author contribution

MAF and RAS designed the experiment. MAF extracted DNA and RNA from the reactor material and prepared 16S Amplicon libraries. Sequencing of the 16S amplicon pools as well as rRNA depletion and preparation of metatranscriptomic libraries and sequencing was performed by SK at the MPI Plön. Data were bioinformatically analysed by MAF. SR and UD provided methane production rates. MAF discussed and interpreted the data with input from SG, SR, UD, WRS and RAS. MAF wrote the manuscript with input from all authors. All authors approved the final version of the manuscript.

## Availability of data and material

Where submitted to NCBI, accession number: SRS2630618 to SRS2630660 with the exception of SRS2630622. The assignment of the individual samples to the respective bacterial and archaeal 16S rRNA gene amplicon and metatranscriptomic datasets is summarized in Table S3. Metagenomic sequences from the same biogas reactor are accessible under PRJNA301928 (http://www.ncbi.nlm.nih.gov/bioproject/PRJNA301928).

## Supporting information


**Fig. S1.** Community composition observed in the metatranscriptome for the bacterial (A) and archaeal (B) community based on the Kaiju analysis.

**Fig. S2.** Observed completeness of the central carbon metabolism as summarized by the KEGG map 1200.

**Fig. S3.** Normalized coverage of high‐quality genomic bins from the metagenomes of the biogas reactor as discussed in Güllert et al. (2016).

**Table S1**. Normalized coverage of the 20 high‐quality bins form Güllert et al. (2016) as observed in the 6 analysed metatranscriptome samples.

**Table S2**. 20 most abundant transcripts within eleven high‐quality bins showing high transcriptional activity and high variance over time.

**Table S3.** Assignment of the individual samples to the datasets submitted to NCBI with their respective SRS number.
