# Supplementary figures and images for: Long‐term investigation of microbial community composition and transcription patterns in a biogas plant undergoing ammonia crisis
Source: Microb Biotechnol. 2018 Oct 31;12(2):305–23. doi: 10.1111/1751-7915.13313 (PMC6390037; doi:10.1111/1751-7915.13313)

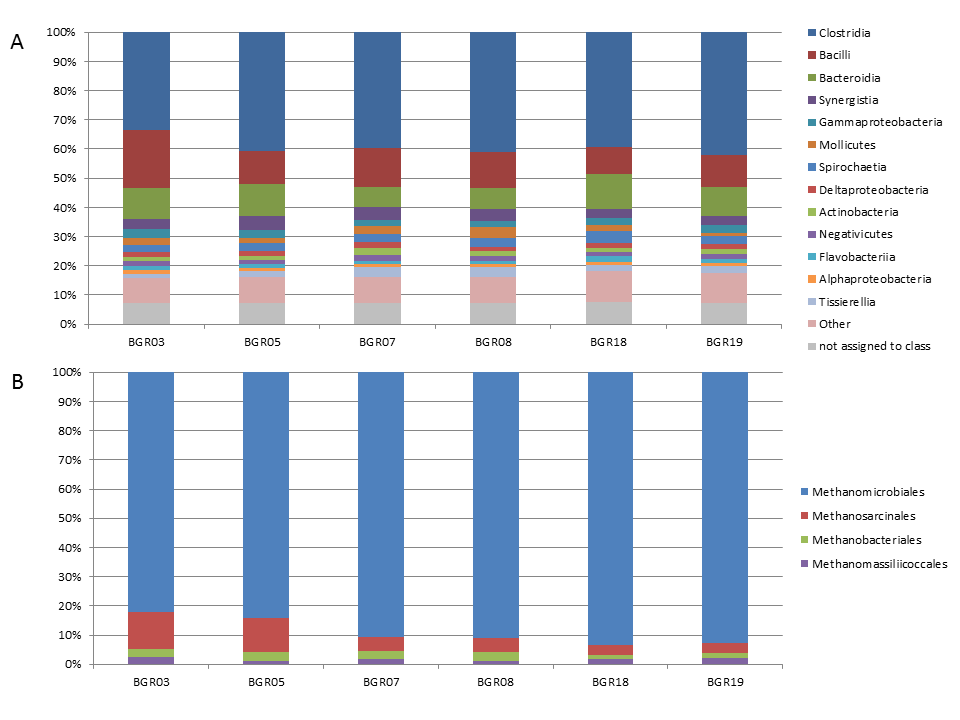

Supplement: Supplementary file 1 — Fig. S1. Community composition observed in the metatranscriptome for the bacterial (A) and archaeal (B) community based on the Kaiju analysis. [file MBT2-12-305-s001.tif]

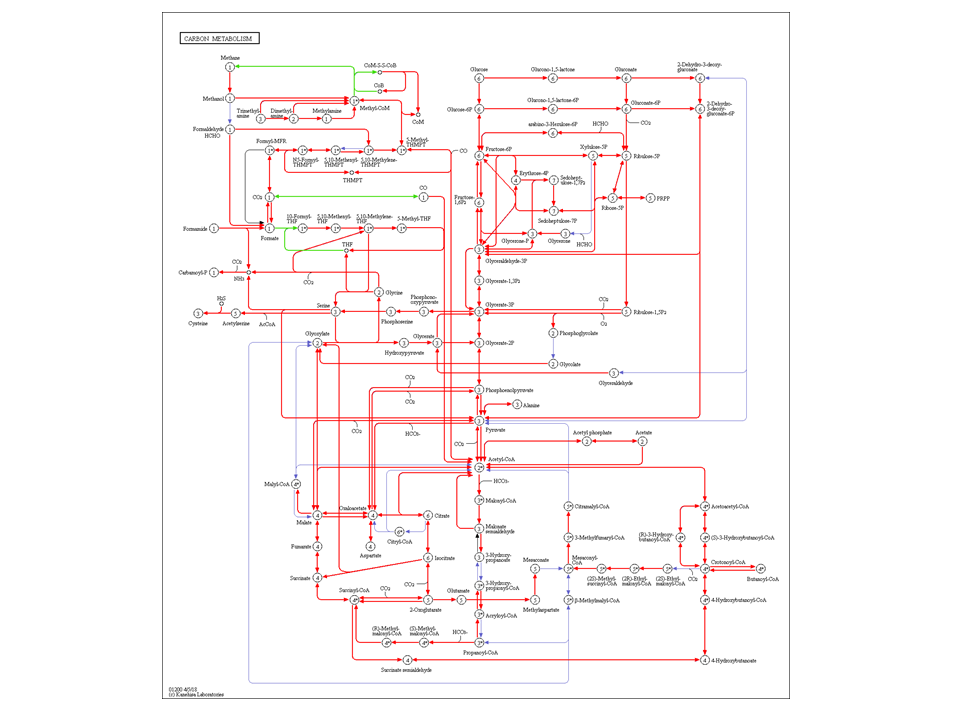

Supplement: Supplementary file 2 — Fig. S2. Observed completeness of the central carbon metabolism as summarized by the KEGG map 1200. [file MBT2-12-305-s002.tif]

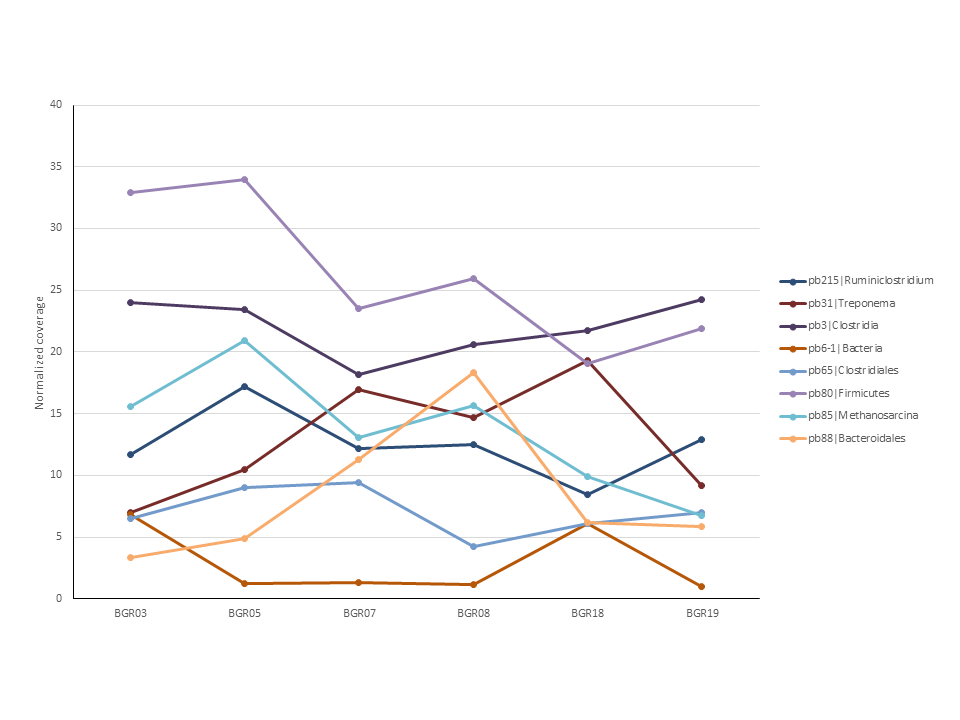

Supplement: Supplementary file 3 — Fig. S3. Normalized coverage of high‐quality genomic bins from the metagenomes of the biogas reactor as discussed in Güllert et al. (2016). [file MBT2-12-305-s003.tif]
